# Determinants of Meningococcal ACWY vaccination in adolescents in the US: completion and compliance with the CDC recommendations

**DOI:** 10.1080/21645515.2019.1632679

**Published:** 2019-08-16

**Authors:** Wendy Y. Cheng, Rose Chang, Patricia Novy, Cristi O’Connor, Mei Sheng Duh, Cosmina S. Hogea

**Affiliations:** a Analysis Group, INC., Boston, MA, USA; b GSK, Philadelphia, PA, USA

**Keywords:** Neisseria meningitidis, MenACWY, vaccine series completion, ACIP schedule, adolescents

## Abstract

Since 2011, the Advisory Committee on Immunization Practices (ACIP) guidelines for routine MenACWY vaccination in the US include a primary dose before age 16 y, preferably at ages 11-12 y, with a booster dose at age 16 y. Data on rates and drivers of meningococcal vaccination completion (receipt of both doses) and compliance with recommendations (receipt of primary dose at ages 11-12 y followed by booster at 16 y) down to state-level are limited.

This study evaluated rates and determinants of MenACWY vaccination completion and compliance in adolescents aged 17 y based on data from the annual National Immunization Survey-Teen between 2011 and 2016. Individual- and state-level determinants of completion and compliance were assessed using uni-level and multi-level multivariable regression models. Average national rates were 23.2% and 12.1% for completion and compliance, respectively, with large inter-state variation observed (completion: 8.7–39.7%; compliance: 3.1–26.2%). Beyond the state of residence, factors significantly associated with a higher likelihood of both completion and compliance included being male, up-to-date on other routine vaccines, having private or hospital-based vaccine providers (vs. public) and having >1 child in the household. Factors specifically associated with completion included having >1 annual health-care visit and presence of a booster-dose vaccine mandate, while a history of asthma and high-risk health conditions had a positive association with compliance. State-level determinants of completion and compliance included pediatricians-to-children ratio and the proportion of Immunization Information System use among adolescents, respectively. Outcomes of this study may help guide clinical, policy and educational interventions to further increase MenACWY completion rates and reduce disparities in vaccination.

## Introduction

Meningococcal disease is a rare but severe illness caused by *Neisseria meningitidis* bacteria. Due to the rapid onset of disease, high case-fatality rate, substantial long-term sequelae among survivors, and the potential for outbreaks, prevention of meningococcal disease remains a public health priority.^1,2^ At least 12 serogroups of *Neisseria meningitidis* have been identified, among which serogroups A, B, C, W, and Y account for nearly all meningococcal disease burden.

Vaccination has proven to be an effective strategy to prevent meningococcal disease.^3^ In 2005, the first quadrivalent meningococcal conjugate vaccine against serogroups A, C, W, and Y (MenACWY) was licensed and recommended by the Advisory Committee on Immunization Practices (ACIP) for routine use in healthy adolescents aged 11–12 years.^4^ In October 2010, with evidence of waning immunity after a single dose of the vaccine the ACIP updated its recommendation to include a booster dose such that the current routine vaccination schedule for healthy adolescents comprises a primary dose at ages 11–12 years and a booster dose at age 16 years.^1,5,6^

From 1996 through to 2015, the incidence of meningococcal disease declined in the US.^7^ Despite this, adolescents and young adults are still at risk of infection and outbreaks of the meningococcal disease continue to occur.^8^ Meningococcal carriage is frequent, with adolescents being the main reservoir,^9,10^ while infection dynamics remains poorly understood.^11^ Globally, increasing trends in serogroup W disease incidence in recent years have been reported.^9,12^ Recent data point to a significant relative burden of serogroups C and Y in adolescents compared to other serogroups; MacNeil et al. speculate that this is likely associated with adolescents either not receiving the MenACWY vaccine or not receiving the MenACWY booster dose in late adolescence.^13^ Adherence to current ACIP recommendations for vaccination constitutes a critical aspect for public health. In 2016, the Centers for Disease Control and Prevention (CDC) reported that while 82.2% of adolescents aged 13 to 17 years received the primary dose, only 39.1% of adolescents aged 17 years received both doses of MenACWY.^14^

The CDC-reported vaccination coverage is estimated from the National Immunization Survey-Teen (NIS-Teen).^15^ To date, the published MenACWY coverage estimates reflect the adolescent age at interview. Vaccination completion (i.e., receipt of primary dose at ages 11–15 y followed by booster dose on or after age 16 y) and compliance to the ACIP recommended schedule based on age at receipt of each dose have not been previously reported. Factors associated with uptake of the MenACWY vaccines during adolescence need further understanding.^16^ While the 82.2% national one-dose coverage reported in 2016 among adolescents aged 13–17 years at interview was relatively high, estimates suggest large differences across states with coverage ranging from 54.2% to 96.4%.^14^ Identifying factors associated with vaccination and adherence to recommendations is important for increasing immunization rates across the board, reducing disparities and ensuring adequate protection against vaccine-preventable diseases.^17^ Information is largely missing regarding determinants of routine MenACWY vaccination in healthy adolescents in the US and subsequent completion and compliance in-line with recommendations.

This study aimed to address this gap and provide a systematic comprehensive analysis of MenACWY primary and booster dose completion and adherence to the ACIP-recommended schedule (compliance) for healthy adolescents down to state level in the US, based on combined NIS-Teen data from years 2011 through 2016. Such evidence can help inform the design and implementation of targeted public health interventions aimed to further increase MenACWY vaccine completion and improve adherence to the ACIP recommended schedule. Primary and booster dose completion and compliance with vaccination guidelines are critical for timely and proper immunization to help prevent meningococcal disease.^16^

## Materials and methods

The study combined six years of NIS-Teen data from 2011 to 2016. The NIS-Teen is an annual survey consisting of a household survey and a provider questionnaire. The household survey collects individual-level data reported by the adolescent’s parent or legal guardian such as the adolescent’s household characteristics, health history, and socio-economic factors. The provider questionnaire collects individual-level data from the vaccination provider, including the adolescent’s vaccination history since birth.^18^ A complete list of individual-level determinants considered, including socio-demographic, economic and health-care characteristics, is provided in Supplementary Table 1. State-level determinants, such as MenACWY education mandates, health-care expenditures, and insurance coverage, were derived from the Kaiser Family Foundation data, the CDC, and the Immunization Action Coalition; a full list is included in Supplementary Table 2.^19–21^

Among the potential state-level variables identified from the Kaiser Family Foundation data, the CDC, and the Immunization Action Coalition, only those that were actionable and non-redundant with any other state- or individual-level variables, were included in the analysis. Of note, the MenACWY primary and booster dose vaccine mandate variables were coded at individual level for simplicity, to easily account for the presence/absence of a mandate in the adolescents’ state of residence at the time they were of the qualifying age since the vaccine mandates varied by survey year within each state during the study period. Thus, an adolescent residing in a state that implemented a MenACWY vaccine mandate for elementary and secondary schools by the time he/she was 15 years of age for the primary dose and 17 years of age for the booster dose, would be attributed a “yes” value for this variable (and a “no” value in case of non-exposure to the mandate). By contrast, MenACWY education mandate variable was considered directly at the state level since no related changes of status occurred during the 2011–2016 time period.

### Study population

The current study included adolescents who were 17 years of age at the time of the household survey and: (1) completed NIS-Teen household surveys; (2) had adequate provider data (APD); (3) lived in non-institutionalized households in the 50 US states or the District of Columbia. Starting in 2014, the NIS-Teen defined an adolescent’s vaccination record as having APD if that adolescent had vaccination history data from one or more of the named vaccination providers or if the parent reported that the adolescent was completely unvaccinated. This updated definition was applied retroactively to data from 2011 to 2013 for consistency. Adolescents were excluded if they received the primary dose of meningococcal-containing vaccine prior to 11 years of age, the earliest age recommended by ACIP in the general adolescent population.

### Sampling weights

Sampling weights specific to each of the survey years were provided by the NIS-Teen datasets and were applied to the analyses to represent the target population. These weights adjusted for factors including household survey non-response rate, provider questionnaire non-response rate, and households with multiple telephone lines or no telephone service. Following guidance from the NIS-Teen, when pooling data across survey years, revised sampling weights were calculated to obtain accurately weighted estimates. These revised weights were derived by dividing each individual’s sampling weight by the total number of survey years.^22^

### Study outcomes

Completion was defined as receipt of the primary dose of MenACWY vaccine at age 11–15 years and a booster dose at age 16 years or older. Compliance with ACIP recommendations was defined as receipt of the primary MenACWY dose at age 11–12 years followed by a booster dose at age 16 years. The assessment of vaccine completion and compliance in this study relied on age at vaccination rather than age at survey administration.

### Statistical analyses

Rates of MenACWY vaccine completion and compliance with ACIP recommendations in adolescents age 17 years were estimated at the national and state level. These estimates are based on multiple years of data and represent a weighted average over the 2011–2016 time period.

Uni-level multivariable logistic regression models were first used to assess the individual-level determinants of both completion and compliance. Multi-level multivariable regression models were then fitted to identify state-level determinants. This method accounts for the clustered nature of the data (i.e., individuals nested within states) while also allowing for the examination of individual-level and state-level variables associated with the outcomes of interest. Multi-level modeling is commonly used in public health research and has been used previously in studies conducted with the NIS-Teen data.^23,24^ Collinearity between determinants was assessed. When conducting multi-level analysis, a backward elimination strategy was applied and a commonly accepted scaled-weight approach was utilized such that the new weights sum to the cluster sample size rather than the target population.^25^ Results are presented as adjusted odds ratio (AOR) estimates and 95% confidence intervals (CI) for all determinants. In addition, the following measures of variation were computed: state-level variance, intraclass correlation coefficient (ICC) (i.e., the proportion of observed variation in the outcome attributable to the effect of clustering by state), and median odds ratio (MOR) (i.e., the magnitude of the effect of clustering by state).

All analyses were conducted using SAS Enterprise Guide software version 7.1 (SAS Institute, Cary, NC). A two-sided alpha error of 0.05 were used to determine statistical significance.

## Results

The study inclusion criteria were met by a total of 22,928 adolescents, representing 3,948,025 adolescents aged 17 years upon weighting (Table 1).

**Table 1.** Estimated MenACWY vaccination completion and compliance rates by selected characteristics among adolescents 17 years of age^a.^

			MenACWY^b^	
			Completion^c^ rate	Compliance^d^ rate
	Unweighted, *N*	Weighted, *N*	*% (95% CI)*	*% (95% CI)*
**Overall**	22,928	3,948,025	23.2 (22.1, 24.2)	12.1 (11.3, 12.9)
**Survey year^e^**				
2011	4,624	687,110	4.4 (3.4, 5.4)	0.8 (0.1, 1.5)
2012	3,707	678,095	15.0 (12.8, 17.3)	3.4 (2.5, 4.2)
2013	3,325	627,244	21.1 (18.6, 23.6)	10.4 (8.4, 12.3)
2014	3,769	664,937	27.9 (25.2, 30.6)	15.9 (13.8, 18.0)
2015	3,882	639,303	32.5 (29.9, 35.1)	19.2 (17.0, 21.5)
2016	3,621	651,336	39.3 (36.4, 42.3)	23.9 (21.2, 26.6)
Demographic characteristics				
**Gender**				
Male	12,050	2,046,554	23.2 (21.8, 24.6)	12.2 (11.2, 13.3)
Female	10,878	1,901,471	23.1 (21.6, 24.6)	11.9 (10.7, 13.1)
**Race/ethnicity**				
Hispanic	3,351	800,971	24.1 (21.1, 27.1)	13.7 (11.2, 16.1)
Non-Hispanic White	15,197	2,258,647	22.0 (20.9, 23.1)	11.6 (10.8, 12.5)
Non-Hispanic Black	2,222	543,138	25.6 (22.5, 28.7)	11.6 (9.6, 13.7)
Non-Hispanic other	2,158	345,269	24.6 (21.3, 27.9)	12.1 (9.5, 14.7)
**Census region**				
Northeast	4,582	662,635	28.7 (26.7, 30.6)	16.1 (14.5, 17.7)
Midwest	5,016	845,413	24.7 (22.9, 26.5)	11.2 (9.9, 12.5)
South	8,142	1,497,619	19.6 (18.2, 21.0)	10.4 (9.3, 11.5)
West	5,188	942,358	23.5 (20.6, 26.5)	12.7 (10.4, 15.1)
**Type of health insurance**				
Private insurance	14,259	2,170,909	24.8 (23.5, 26.1)	12.9 (11.8, 14.0)
Any Medicaid	5,547	1,184,107	23.8 (21.8, 25.8)	12.2 (10.8, 13.7)
Other insurance^f^	1,753	295,075	20.9 (16.8, 24.9)	12.7 (9.4, 16.0)
Uninsured	1,296	280,247	11.1 (7.7, 14.5)	5.2 (2.6, 7.9)
Maternal characteristics				
**Mother’s marital status**				
Married	16,664	2,581,104	24.1 (22.9, 25.3)	12.5 (11.5, 13.5)
Not married	6,264	1,366,921	21.4 (19.6, 23.1)	11.4 (10.0, 12.7)
Household characteristics				
**Number of children <18 in household**				
1	11,711	1,647,812	20.9 (19.6, 22.2)	10.7 (9.7, 11.7)
2–3	9,493	1,891,276	25.8 (24.2, 27.4)	13.5 (12.2, 14.8)
≥4	1,724	408,938	19.9 (16.7, 23.2)	11.2 (8.2, 14.1)
**Family income**				
≤$30,000	4,709	1,043,918	20.0 (18.0, 22.1)	10.3 (8.9, 11.8)
$30,001-$75,000	6,577	1,143,653	21.0 (19.2, 22.9)	10.6 (9.1, 12.1)
>$75,000	10,340	1,472,076	27.0 (25.4, 28.6)	14.3 (13.0, 15.6)
Healthcare history				
**Number of visits to healthcare professional in the past year**				
None	3,322	649,400	14.5 (12.2, 16.9)	7.8 (5.9, 9.7)
1	6,084	1,084,350	24.8 (22.8, 26.9)	13.1 (11.5, 14.7)
2–5	10,906	1,801,435	25.2 (23.7, 26.7)	12.9 (11.7, 14.1)
≥6	2,460	380,886	23.8 (20.6, 27.0)	12.9 (10.2, 15.5)
**Whether teen had a 11–12-year-old well-child exam**				
Yes	19,761	3,395,589	24.9 (23.7, 26.0)	13.0 (12.1, 13.9)
No	1,414	232,132	11.7 (8.5, 15.0)	5.2 (2.6, 7.8)
**Asthma history**				
Yes	4,790	830,756	25.9 (23.6, 28.3)	15.3 (13.1, 17.4)
No	18,101	3,108,336	22.5 (21.3, 23.6)	11.3 (10.4, 12.1)
**Any high-risk health conditions**^g^				
Yes	1,808	310,766	25.5 (21.3, 29.6)	15.5 (11.6, 19.5)
No	21,099	3,633,744	23.0 (21.9, 24.0)	11.8 (11.0, 12.6)
**Any high-risk health conditions among household members^g^**				
Yes	8,916	1,504,742	21.6 (20.1, 23.1)	10.8 (9.7, 11.9)
No	13,983	2,439,810	24.1 (22.7, 25.5)	12.9 (11.8, 13.9)
Provider information^h^				
**Facility type of vaccine providers**				
Public	3,502	605,888	14.3 (12.0, 16.6)	6.8 (5.3, 8.3)
Private	10,345	1,903,851	26.6 (25.1, 28.1)	14.3 (13.1, 15.6)
Hospital	2,209	322,172	24.9 (21.6, 28.2)	14.2 (11.4, 17.0)
Other/mixed/unknown	6,748	1,104,180	21.8 (19.9, 23.7)	10.7 (9.2, 12.1)
**Whether teen’s providers report vaccinations to immunization registry**				
No providers	3,498	622,055	18.9 (16.7, 21.2)	10.1 (8.2, 11.9)
Some providers	3,041	481,097	21.8 (19.0, 24.6)	10.3 (8.0, 12.7)
All providers	12,476	2,117,901	25.5 (24.1, 26.9)	13.7 (12.6, 14.8)
Unknown	3,789	715,038	21.1 (18.5, 23.7)	10.4 (8.4, 12.3)
Up-to-date on other vaccines^i^				
**Hepatitis A**				
Yes	10,830	1,930,323	34.7 (32.9, 36.4)	18.9 (17.5, 20.4)
No	12,098	2,017,703	12.1 (11.2, 13.1)	5.6 (4.9, 6.2)
**Hepatitis B**				
Yes	20,521	3,549,390	24.9 (23.8, 26.0)	13.0 (12.1, 13.9)
No	2,407	398,635	7.7 (5.7, 9.7)	4.0 (2.2, 5.7)
**Varicella**				
Yes	16,545	2,951,658	27.8 (26.5, 29.1)	14.8 (13.7, 15.8)
No	6,383	996,367	9.4 (8.0, 10.8)	4.2 (3.1, 5.3)
**HPV**				
Yes	7,616	1,278,842	40.4 (38.3, 42.5)	23.2 (21.4, 24.9)
No	15,312	2,669,184	14.9 (13.9, 15.9)	6.8 (6.0, 7.6)
**Pneumococcal polysaccharide**				
Yes	979	181,700	39.3 (32.9, 45.7)	25.0 (18.9, 31.2)
No	21,949	3,766,326	22.4 (21.4, 23.4)	11.5 (10.7, 12.2)
**Tdap**				
Yes	18,638	3,222,910	27.2 (26.0, 28.4)	14.4 (13.4, 15.3)
No	4,290	725,115	5.3 (4.3, 6.4)	1.9 (1.4, 2.5)
Vaccine mandates^j^				
**Residence in a state with one-dose vaccination mandate by age 15**				
Yes	7,428	1,156,163	30.3 (28.6, 32.0)	17.8 (16.4, 19.2)
No	15,500	2,791,862	20.2 (19.0, 21.5)	9.7 (8.8, 10.7)
**Residence in a state with booster dose vaccination mandate by age 17**				
Yes	1,758	249,173	40.6 (37.3, 44.0)	17.0 (14.5, 19.5)
No	21,170	3,698,852	22.0 (20.9, 23.0)	11.8 (10.9, 12.6)

ACIP: Advisory Committee on Immunization Practices; CI: confidence interval; HPV: human papillomavirus; Tdap: tetanus-diphtheria-acellular-pertussis vaccine

Footnotes:

a. Includes adolescents who were age 17 at the time of household survey with adequate provider data. Adolescents vaccinated before age 11 were excluded.

b. All estimates are presented as 6-year averages for 2011–2016.

c. Completion is defined as receipt of the vaccine primary dose at ages 11–15 and booster dose at or after age 16.

d. Compliance is defined as receipt of the vaccine primary dose at ages 11–12 and booster dose at age 16.

e. The weighted N for survey year sums to the overall population total due to the use of the revised sampling weights.

f. Other insurance includes Children’s Health Insurance Program, Indian Health Service, and health insurance provided by the military.

g. High-risk health conditions include lung conditions other than asthma, heart conditions, diabetes, kidney conditions, sickle cell anemia or other anemia, or a weakened immune system caused by a chronic illness or by medicines taken for a chronic illness.

h. Provider-reported data is collected from the provider-immunization history questionnaire.

i. Up-to-date on other vaccines excludes any vaccinations received after the telephone survey date and is defined as having the following: hepatitis A: 2+ hepatitis-A-containing shots; hepatitis B: 2+ hepatitis B 1.0 milliliter RECOMBIVAX shots, or 3+ any combination of hepatitis-b-containing shots; varicella: 1+ varicella-containing shot at 12+ months of age; HPV: 3+ human papillomavirus shots; Tdap: 1+ Tdap-only shot since age 10 years.

j. Variables created using data from the Immunization Action Coalition.

### MenACWY vaccination completion and compliance rates: descriptive statistics

During 2011–2016, the average national rate of MenACWY vaccine primary and booster dose completion was 23.2%, with an increase over time (4.4% in 2011 to 39.3% in 2016). During the same time period, the average rate of compliance with ACIP recommendations for MenACWY vaccination was 12.1% (increasing from 0.8% in 2011 to 23.9% in 2016) (Table 1).

**Figure 1.**
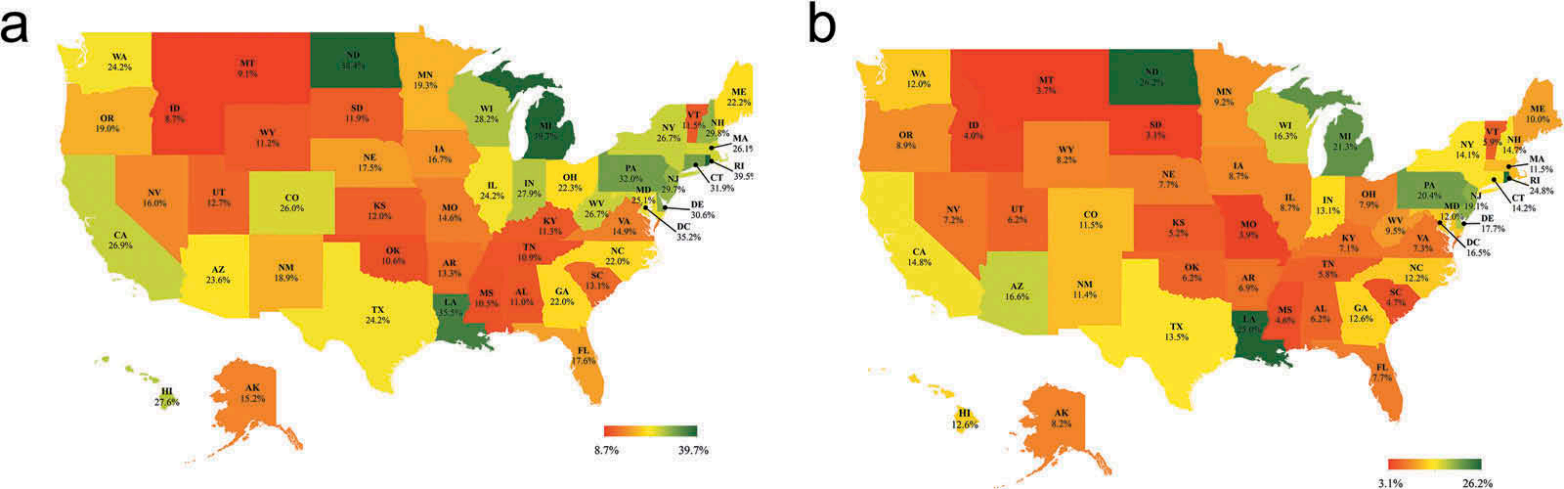
State-specific completion^a^ (a) and compliance^b^ (b) rates^c^ for MenACWY vaccination, 2011–2016^d.^ a. Completion is defined as receipt of the vaccine primary dose at ages 11–15 and booster dose at or after age 16. b. Compliance is defined as receipt of the vaccine primary dose at ages 11–12 and the booster dose at age 16. c. Includes adolescents who were age 17 at the time of household survey with adequate provider data. Adolescents who received a meningococcal-containing vaccination before age 11 were excluded. d. Estimates are presented as 6-year averages for 2011–2016.

Large interstate variations were observed for both completion, varying from 8.7% in Idaho to 39.7% in Michigan (Figure 1(a)), and compliance rates (3.1% in South Dakota to 26.2% in North Dakota) (Figure 1(b)).

Notable differences in vaccination were present across different characteristics described in Table 1. MenACWY completion and compliance were higher for adolescents living in the Northeast and across states with existing vaccination mandates. Adolescents with a family income >$75,000, who had an 11–12-year-old well-child exam or who were up-to-date on other vaccines including hepatitis A, hepatitis B, varicella, human papillomavirus (HPV), pneumococcal polysaccharide, and tetanus-diphtheria-acellular pertussis (Tdap), had higher completion and compliance rates. Lower completion and compliance rates were observed in adolescents having no health insurance, no visit to a health-care professional in the past year, receiving vaccines in a public facility, and having a vaccine provider who does not report vaccinations to the immunization registry.

### Determinants of completion and compliance: multivariable analyses

#### MenACWY vaccination completion

Findings from uni-level multivariable logistic regression modeling indicate that the odds of completing both the primary and booster dose increased with time since recommendation. Higher odds of series completion were also found among adolescents who were non-Hispanic Black (AOR = 1.38, *p* = .006), had married mothers (AOR = 1.23, *p* = .023), had 2–3 children <18 years of age in the household (AOR = 1.22, *p* = .005), and had a family income >$75,000 (AOR = 1.31, *p* = .013). Adolescents of female gender (AOR = 0.62, *p* < .001) and with household members with any high-risk health conditions (AOR = 0.84, *p* = .016) had lower odds of series completion. Healthcare-related determinants associated with completion were the number of visits to health-care professionals in the past year, whether the adolescent had an 11–12-year-old well-child exam, presence of household members with any high-risk health condition, facility type of vaccine providers, whether the adolescent’s providers reported vaccinations to an immunization registry, and being up-to-date on other vaccines. Specifically, adolescents who had two or more visits to health-care professionals in the past year (2–5 visits: AOR = 1.43, *p* = .003; ≥6 visits: AOR = 1.47, *p* = .016), had an 11–12-year-old well-child exam (AOR = 1.48, *p* = .039), received vaccines at private, hospital, or other/mixed/unknown facilities (Private: AOR = 1.69, *p* < .001; Hospital: AOR = 1.63, *p* = .002; Other/mixed/unknown: AOR = 1.58, *p* = .001), had all their providers reporting vaccinations to immunization registry (AOR = 1.43, *p* = .001), were up-to-date with their hepatitis A (AOR = 2.31, *p* < .001), hepatitis B (AOR = 1.67, *p* = .005), varicella (AOR = 1.43, *p* = .001), HPV (AOR = 2.60, *p* < .001), and Tdap (AOR = 3.28, *p* < .001) vaccines had higher odds of series completion. Notably, adolescents residing in states with a vaccination mandate for the booster dose (AOR = 2.03, *p* < .001) had a significantly higher likelihood of completion (Table 2).

**Table 2.** Uni-level multivariable logistic regression model for MenACWY primary and booster dose completion and compliance among adolescents 17 years of age^a,b.^

	Completion rate^c^		Compliance rate^d^	
	Adjusted odds ratio (95% CI)	P-value	Adjusted odds ratio (95% CI)	P-value
Demographic characteristics				
**Survey year (ref.: 2011)**				
2012	3.55 (2.54–4.97)	**<0.001**	3.85 (1.45–10.2)	**0.007**
2013	4.90 (3.53–6.81)	**<0.001**	12.5 (4.77–32.6)	**<0.001**
2014	5.82 (4.23–8.01)	**<0.001**	17.4 (6.70–45.3)	**<0.001**
2015	6.73 (4.89–9.26)	**<0.001**	21.3 (8.27–54.9)	**<0.001**
2016	7.89 (5.69–11.0)	**<0.001**	27.6 (10.6–71.7)	**<0.001**
**Gender (ref.: Male)**				
Female	0.62 (0.53–0.72)	**<0.001**	0.65 (0.54–0.77)	**<0.001**
**Race/ethnicity (ref.: Non-Hispanic White)**				
Non-Hispanic Black	1.38 (1.10–1.74)	**0.006**	–	
Non-Hispanic other	1.20 (0.94–1.53)	0.148	–	
Hispanic	1.13 (0.91–1.42)	0.271	–	
**Type of health insurance (ref.: Private insurance)**				
Any Medicaid	–		0.90 (0.74–1.10)	0.315
Other insurance^f^	–		1.35 (0.99–1.84)	0.057
Uninsured	–		0.60 (0.33–1.09)	0.092
Maternal characteristics				
**Mother’s marital status (ref.: Not married)**				
Married	1.23 (1.03–1.47)	**0.023**	–	
Household characteristics				
**Number of children <18 in household (ref.: 1)**				
2–3	1.22 (1.06–1.41)	**0.005**	1.25 (1.05–1.48)	**0.010**
≥4	1.11 (0.84–1.46)	0.480	1.22 (0.86–1.73)	0.271
**Family income (ref.: ≤$30,000)**				
$30,001-$75,000	1.13 (0.92–1.39)	0.247	–	
>$75,000	1.31 (1.06–1.62)	**0.013**	–	
Healthcare history				
**Number of visits to healthcare professional in the past year (ref.: None)**				
1	1.24 (0.96–1.61)	0.096	–	
2–5	1.43 (1.13–1.82)	**0.003**	–	
≥6	1.47 (1.07–2.02)	**0.016**	–	
**Whether teen had a 11–12-year-old well-child exam (ref.: No)**				
Yes	1.48 (1.02–2.16)	**0.039**	–	
**Asthma history (ref.: No)**				
Yes	–		1.33 (1.09–1.62)	**0.005**
**Any high-risk health conditions^g^ (ref.: No)**				
Yes	–		1.42 (1.02–1.96)	**0.036**
**Any high-risk health conditions among household members^g^ (ref.: No)**				
Yes	0.84 (0.73–0.97)	**0.016**	0.79 (0.66–0.93)	**0.006**
Provider information^g^				
**Facility type of vaccine providers (ref.: Public)**				
Private	1.69 (1.30–2.19)	**<0.001**	1.92 (1.43–2.58)	**<0.001**
Hospital	1.63 (1.19–2.23)	**0.002**	1.80 (1.24–2.63)	**0.002**
Other/mixed/unknown	1.58 (1.19–2.09)	**0.001**	1.46 (1.06–2.02)	**0.022**
**Whether teen’s providers report vaccinations to immunization registry (ref.: No providers)**				
Some providers	1.13 (0.83–1.55)	0.426	–	
All providers	1.43 (1.15–1.77)	**0.001**	–	
Unknown	1.15 (0.88–1.50)	0.302	–	
Up-to-date on other vaccines^h^				
**Hepatitis A (ref.: No)**				
Yes	2.31 (1.99–2.69)	**<0.001**	2.21 (1.82–2.69)	**<0.001**
**Hepatitis B (ref.: No)**				
Yes	1.67 (1.17–2.40)	**0.005**	–	
**Varicella (ref.: No)**				
Yes	1.43 (1.15–1.77)	**0.001**	–	
**HPV (ref.: No)**				
Yes	2.60 (2.22–3.04)	**<0.001**	2.88 (2.39–3.47)	**<0.001**
**Tdap (ref.: No)**				
Yes	3.28 (2.42–4.44)	**<0.001**	4.37 (3.09–6.18)	**<0.001**
Vaccine mandate				
**Residence in a state with booster dose vaccination mandate by age 17^i^ (ref.: No)**				
Yes	2.03 (1.45–2.83)	**<0.001**	–	

ACIP: Advisory Committee on Immunization Practices; CI: confidence interval; HPV: human papillomavirus; MenACWY: meningococcal conjugate vaccine; Tdap: tetanus-diphtheria-acellular-pertussis vaccine

**Figure 2.**
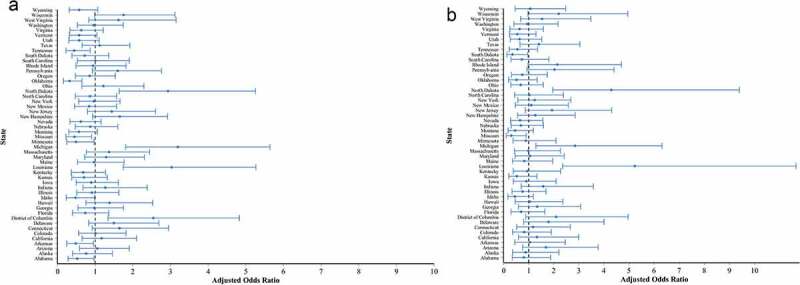
Likelihood^a^ of MenACWY vaccination completion (a) and compliance (b) by state of residence (reference: Mississippi) based on multivariable logistic regression. ^a^In multivariable analysis, the overall categorical variable “state of residence” had a significant effect on the likelihood of MenACWY vaccine completion and compliance. The adjusted odds ratio of each state as compared to the reference (i.e., Mississippi) is shown with 95% confidence interval.

The model also included state of residence (data presented in Figure 2).

Footnotes:

a. Includes adolescents who were age 17 at the time of household survey with adequate provider data. Adolescents vaccinated before age 11 were excluded.

b. All estimates are presented as 6-year averages for 2011–2016. Backward elimination was used for model selection. Bold text indicates *p* < 0.05.

c. Completion is defined as receipt of the vaccine primary dose at ages 11–15 and booster dose at or after age 16.

d. Compliance is defined as receipt of the vaccine primary dose at ages 11–12 and the booster dose at age 16.

e. Other insurance includes Children’s Health Insurance Program, Indian Health Service, and health insurance provided by the military.

f. High-risk health conditions include lung conditions other than asthma, heart conditions, diabetes, kidney conditions, sickle cell anemia or other anemia, or a weakened immune system caused by a chronic illness or by medicines taken for a chronic illness.

g. Provider-reported data is collected from the provider-immunization history questionnaire.

h. Up-to-date on other vaccines excludes any vaccinations received after the telephone survey date and is defined as having the following: hepatitis A: 2+ hepatitis-A-containing shots; hepatitis B: 2+ hepatitis B 1.0 milliliter RECOMBIVAX shots, or 3+ any combination of hepatitis-b-containing shots; varicella: 1+ varicella-containing shot at 12+ months of age; HPV: 3+ human papillomavirus shots; Tdap: 1+ Tdap-only shot since age 10 years.

i. The MenACWY booster mandate variable was created using data from the Immunization Action Coalition.

After controlling for demographic and clinical characteristics as well as provider characteristics, large variations remained in the likelihood of MenACWY primary and booster dose completion across states of residence (Figure 2(a)).

Similar patterns of associations between individual-level determinants and MenACWY vaccine completion were observed in the multi-level model (Table 3). Following backward elimination, one state-level determinant was selected: adolescents residing in states with higher pediatrician density had a significantly higher likelihood of MenACWY completion compared to individuals in states with lower pediatrician density (AOR = 1.69, *p* = .007). Adjusting for individual-level determinants decreased the state-level variance from 0.27 to 0.25 and MOR from 1.64 to 1.61. Further, adjusting for the significant state-level determinant (the number of pediatricians per 10,000 population ages 0–18) decreased the state-level variance by 16.1% from 0.25 to 0.21 and the MOR from 1.61 to 1.54. The MOR suggests that residual heterogeneity between states was of greater relevance for understanding the likelihood of MenACWY completion compared to most of the individual-level determinants (ORs ranging from 0.63 to 1.56), excluding being up-to-date with other vaccines and survey year, which had much higher ORs. The MOR in the multi-level model remained greater than 1, suggesting that the differences in the odds across states could not be fully explained by the considered individual-level and state-level determinants.

**Table 3.** Multi-level multivariable logistic regression models for MenACWY receipt of both the primary and booster dose (i.e., completion^a^) among adolescents 17 years of age^b,c.^

	Odds ratio (95% CI)	P-value
**Survey year (ref.: 2011)**		
2012	3.39 (2.79–4.10)	**<0.001**
2013	5.03 (3.77–6.71)	**<0.001**
2014	6.12 (4.77–7.85)	**<0.001**
2015	6.85 (5.33–8.81)	**<0.001**
2016	8.10 (6.31–10.39)	**<0.001**
**Gender (ref.: Male)**		
Female	0.63 (0.56–0.71)	**<0.001**
**Race/ethnicity (ref.: Non-Hispanic White)**		
Non-Hispanic Black	1.30 (1.05–1.61)	**0.016**
Non-Hispanic other	1.37 (1.09–1.71)	**0.006**
Hispanic	1.16 (1.02–1.32)	**0.024**
**Mother’s marital status (ref.: Not married)**		
Married	1.14 (1.01–1.28)	**0.035**
**Number of children <18 in household (ref.: 1)**		
2–3	1.14 (1.03–1.27)	**0.013**
≥4	1.14 (0.97–1.34)	0.102
**Family income (ref.: ≤$30,000)**		
$30,001-$75,000	1.05 (0.92–1.20)	0.487
>$75,000	1.21 (1.02–1.45)	**0.033**
**Number of visits to healthcare professional in the past year (ref.: None)**		
1	1.36 (1.11–1.67)	**0.003**
2–5	1.52 (1.25–1.85)	**<0.001**
≥6	1.44 (1.10–1.88)	**0.008**
**Whether teen had a 11–12-year-old well-child exam (ref.: No)**		
Yes	1.41 (1.14–1.76)	**0.002**
**Any high-risk health conditions among household members^d^ (ref.: No)**		
Yes	0.90 (0.79–1.01)	0.083
**Facility type of vaccine providers (ref.: Public)**		
Private	1.56 (1.29–1.90)	**<0.001**
Hospital	1.42 (1.10–1.83)	**0.007**
Other/mixed/unknown	1.49 (1.24–1.78)	**<0.001**
**Whether teen’s providers report vaccinations to immunization registry (ref.: No providers)**		
Some providers	1.15 (0.86–1.55)	0.354
All providers	1.31 (1.11–1.55)	**0.002**
Unknown	1.11 (0.91–1.34)	0.299
**Up-to-date Hepatitis A^e^ (ref.: No)**		
Yes	2.37 (2.07–2.71)	**<0.001**
**Up-to-date Hepatitis B^e^ (ref.: No)**		
Yes	1.77 (1.16–2.69)	**0.008**
**Up-to-date Varicella^e^ (ref.: No)**		
Yes	1.39 (1.19–1.63)	**<0.001**
**Up-to-date HPV^e^ (ref.: No)**		
Yes	2.77 (2.38–3.21)	**<0.001**
**Up-to-date Tdap^e^ (ref.: No)**		
Yes	3.03 (2.51–3.67)	**<0.001**
**Residence in a state with booster dose vaccination mandate by age 17 (ref.: No)**		
Yes	2.08 (1.48–2.93)	**<0.001**
*Level 2 Predictors*		
**Pediatricians per 10,000 population ages 0–18 (per 10-unit increase) (ref.: 0 to <7.6 (1st quartile))**		
7.6 to <8.9 (2nd quartile)	1.08 (0.75–1.57)	0.664
8.9 to <11.8 (3rd quartile)	1.39 (0.89–2.18)	0.151
11.8 to <56.5 (4th quartile)	1.69 (1.16–2.46)	**0.007**
***Measures of variation or clustering***		
State-level variance (SE)	**0.21 (0.06)**	
ICC^f^	0.059	
MOR	1.54	

CI: confidence interval; HPV: human papillomavirus; ICC: intraclass correlation coefficient; MOR: median odds ratio; MenACWY: meningococcal conjugate vaccine; SE: standard error; Tdap: tetanus-diphtheria-acellular-pertussis vaccine

Footnotes:

a. Completion is defined as receipt of the vaccine primary dose at ages 11–15 and booster dose at or after age 16.

b. Includes adolescents who were age 17 at the time of household survey with adequate provider data. Adolescents who received a meningococcal-containing vaccination before age 11 were excluded.

c. All estimates are presented as 6-year averages for 2011–2016. Backward elimination was used for model selection. Bold text indicates *p* < 0.05.

d. High-risk health conditions include lung conditions other than asthma, heart conditions, diabetes, kidney conditions, sickle cell anemia or other anemia, or a weakened immune system caused by a chronic illness or by medicines taken for a chronic illness.

e. Up-to-date on other vaccines excludes any vaccinations received after the telephone survey date and is defined as having the following: hepatitis A: 2+ hepatitis-A-containing shots; hepatitis B: 2+ hepatitis B 1.0 milliliter RECOMBIVAX shots, or 3+ any combination of hepatitis-b-containing shots; varicella: 1+ varicella-containing shot at 12+ months of age; HPV: 3+ human papillomavirus shots; Tdap: 1+ Tdap-only shot since age 10 years.

f. The ICC from an empty model with no individual or state-level determinants was 0.075.

#### MenACWY vaccination compliance with ACIP recommendations

Likelihood of compliance was also higher among adolescents who had 2–3 other children <18 years of age also living in the household (AOR = 1.25, *p* = .01), but lower among female adolescents (AOR = 0.65, *p* < .001). Certain health-care determinants were associated with higher odds of compliance, including having a history of asthma (AOR = 1.33, *p* = .005), having had any high-risk health condition (AOR = 1.42, *p* = .036), having had private, hospital, or other/mixed/unknown facilities providing vaccines (AORs = 1.46–1.92, all *p* < .022), and being up-to-date on their hepatitis A (AOR = 2.21, *p* < .001), HPV (AOR = 2.88, *p* < .001), and Tdap (AOR = 4.37, *p* < .001) vaccines. Adolescents with household members with any high-risk health conditions (AOR = 0.79, *p* = .006) had lower odds of compliance (Table 2).

Substantial inter-state variability in MenACWY vaccination compliance remained after adjusting for demographic and clinical characteristics (Figure 2(b)).

Largely similar patterns of associations between individual-level determinants and compliance remained in the multi-level models that included state-level determinants (Table 4) compared to the uni-level logistic regression results. In the multi-level model, type of health insurance became significantly associated with compliance, while the association with high-risk health conditions was reduced to non-significance. Two state-level determinants – health-care expenditures on physician/clinical services per capita and use of Immunization Information Systems (IIS) – were selected from the original list of potential state-level determinants included. Every 10% increase in the proportion of adolescents participating in an IIS was significantly associated with an increased likelihood of compliance (AOR = 1.09, *p* = .012), after adjusting for individual-level determinants (Table 4). Individual-level determinants reduced the state-level variance from 0.31 to 0.29 and the MOR by 1.76% from 1.70 to 1.67. Further adjustment for health-care expenditures on physician and clinical services and proportion of IIS use among adolescents lead to a slightly greater decrease in the MOR of 4.79% (from 1.67 to 1.59), and state-level variance of 18.6% (from 0.29 to 0.24). Similar to completion, the MOR also remained greater than 1, indicating that the difference in the odds of compliance across states could also not be fully explained by the individual-level and state-level determinants included.

**Table 4.** Multi-level multivariable logistic regression models for MenACWY vaccination compliance with ACIP recommendation^a^ among adolescents 17 years of age^b,c.^

	Odds ratio (95% CI)	P-value
**Survey year (ref.: 2011)**		
2012	8.45 (4.70–15.22)	**<0.001**
2013	21.4 (12.9–35.7)	**<0.001**
2014	32.5 (18.9–55.8)	**<0.001**
2015	37.7 (21.9–64.8)	**<0.001**
2016	46.1 (27.3–78.0)	**<0.001**
**Gender (ref.: Male)**		
Female	0.67 (0.60–0.76)	**<0.001**
**Type of health insurance (ref.: Private insurance)**		
Any Medicaid	1.05 (0.88–1.26)	0.58
Other insurance^d^	1.48 (1.22–1.79)	**<0.001**
Uninsured	0.58 (0.40–0.85)	**0.005**
**Number of children <18 in household (ref.: 1)**		
2–3	1.12 (1.01–1.24)	**0.031**
≥4	1.05 (0.86–1.28)	0.622
**Asthma history (ref.: No)**		
**Yes**	1.17 (1.00–1.37)	**0.044**
**Any high-risk health conditions^e^ (ref.: No)**		
**Yes**	1.23 (0.98–1.54)	0.08
**Any high-risk health conditions among household members^e^ (ref.: No)**		
Yes	0.84 (0.76–0.94)	**0.002**
**Facility type of vaccine providers (ref.: Public)**		
Private	1.81 (1.48–2.20)	**<0.001**
Hospital	1.53 (1.21–1.93)	**<0.001**
Other/mixed/unknown	1.46 (1.19–1.80)	**<0.001**
**Up-to-date Hepatitis A^f^ (ref.: No)**		
Yes	2.20 (1.87–2.59)	**<0.001**
**Up-to-date HPV^f^ (ref.: No)**		
Yes	3.18 (2.82–3.60)	**<0.001**
**Up-to-date Tdap^f^ (ref.: No)**		
Yes	3.80 (2.75–5.25)	**<0.001**
Healthcare expenditures on physician and clinical services per capita (per $100-unit increase)	1.06 (0.99–1.12)	**0.075**
Proportion of IIS use among adolescents (per 10 percent unit increase)^g^	1.09 (1.02–1.17)	**0.012**
***Measures of variation or clustering***		
State level variance (SE)	0.24 (0.06)	
ICC^h^	0.067	
MOR	1.59	

ACIP: Advisory Committee on Immunization Practices; CI: confidence interval; HPV: human papillomavirus; ICC: intraclass correlation coefficient; IIS: Immunization Information Systems; MOR: median odds ratio; MenACWY: meningococcal conjugate vaccine; SE: standard error; Tdap: tetanus-diphtheria-acellular-pertussis vaccine

Footnotes:

a. Compliance is defined as receipt of the vaccine primary dose at ages 11–12 and the booster dose at age 16.

b. Includes adolescents who were age 17 at the time of household survey with adequate provider data. Adolescents who received a meningococcal-containing vaccination before age 11 were excluded.

c. All estimates are presented as 6-year averages for 2011–2016. Backward elimination was used for model selection. Bold text indicates *p* < 0.05.

d. Other insurance includes Children’s Health Insurance Program, Indian Health Service, and health insurance provided by the military.

e. High-risk health conditions include lung conditions other than asthma, heart conditions, diabetes, kidney conditions, sickle cell anemia or other anemia, or a weakened immune system caused by a chronic illness or by medicines taken for a chronic illness.

f. Up-to-date on other vaccines excludes any vaccinations received after the telephone survey date and is defined as having the following: hepatitis A: 2+ hepatitis-A-containing shots; HPV: 3+ human papillomavirus shots; Tdap: 1+ Tdap-only shot since age 10 years.

g. IIS use is defined as the average percentage of US adolescents 11–17 years participating in an IIS between years 2011-2016.

h. The ICC from an empty model with no individual or state-level determinants was 0.085.

## Discussion

Using the 2011–2016 NIS-Teen data, this study estimated the US national and state rate of MenACWY vaccine primary and booster dose completion and compliance with ACIP recommendation in adolescents, with focus on inter-state variability and determinants of vaccination. The average rates of MenACWY vaccine completion (23.2%) and compliance (12.1%) during the study period were suboptimal and varied across states. The extent to which individual-level determinants including demographics, clinical or provider characteristics, state of residence, and vaccine mandates, as well as state-level determinants such as the number of pediatricians per 10,000 persons aged 0–18 years, state health-care expenditures, education mandates and IIS utilization, were associated with the likelihood of MenACWY vaccination was assessed to further investigate potential determinants of these differences in completion and compliance. While other studies have assessed determinants of vaccination in adolescents,^26,27^ to our knowledge, this is the first study to investigate MenACWY vaccine primary and booster dose completion and compliance with ACIP recommendation using multi-level modeling to determine the impact of state-level factors in addition to individual-level factors.

Various individual-level demographic determinants (family income, race/ethnicity) were associated with MenACWY vaccination completion and compliance. Consistent with published literature, adolescents from higher-income families had better odds of completing both the primary and booster dose during early adolescence.^27^ Non-Hispanic Black adolescents were more likely to complete both doses than non-Hispanic Whites. These findings may be influenced by the Vaccines for Children (VFC) program which aims to close the disparities in healthcare access by providing free recommended vaccinations for children ≤18 years of age, Medicaid-eligible, uninsured, underinsured, or American Indian or Alaska Native.^28^

Clinical and provider characteristics were also important determinants of MenACWY vaccine primary and booster dose completion. Higher frequency of health-care visits in the past year and having an 11–12-year-old well-child exam were associated with a 1.4 to 1.5-fold increase in likelihood for vaccine completion. A higher likelihood of vaccine completion was also influenced by determinants related to healthcare contact including high-risk health conditions. In addition, being up-to-date on other adolescent vaccines, particularly HPV, hepatitis A, and Tdap vaccines, was consistently associated with a higher likelihood of both vaccination outcomes. Furthermore, among all significant individual-level determinants, being up-to-date on these three vaccines had the strongest influence on the likelihood of vaccine completion and compliance. This is in line with previous studies, which have reported that the HPV vaccine-series initiation is significantly associated with Tdap and meningococcal vaccines’.^29,30^

In recent years, an increasing number of states have implemented state mandates for both the primary and booster dose of the MenACWY vaccine, following the ACIP recommendations.^31^ In the multivariable analysis, the presence of the booster-dose mandate before the age of 17 more than doubled the likelihood of completion. These results provide additional support for the importance of state vaccine mandates on MenACWY vaccine primary and booster dose completion. This analysis yielded two noteworthy findings concerning state vaccine mandates. First, the primary dose mandate was not a significant determinant of MenACWY completion. This may partly be explained by the fact that one-dose vaccine recipients were included in the comparison group. Therefore, the impact of the primary dose mandate may have been diluted, resulting in the booster dose mandate being the predominant driver for completion of both vaccine doses. Alternatively, a primary dose mandate might not significantly affect the completion of the booster dose due to differences in its enforcement compared to the enforcement of a booster dose mandate. Second, neither the one-dose mandate nor the booster dose mandate was a significant determinant of vaccine compliance. This finding could potentially be explained by dilution effects due to the inclusion of one-dose recipients, similar to that for completion, and recipients of both doses but not in accordance with ACIP recommendation, in the comparison group; and the relatively low proportion of adolescents who complied with the ACIP vaccination schedule (12.1% overall), who may possess highly different behaviors and drivers to be vaccinated compared to other adolescents.

In addition to vaccine mandates, states may implement education mandates to provide more information on the vaccines. However, in this study, state education mandate as a state-level determinant was not significantly associated with either MenACWY vaccine outcomes. It is possible that the effect of the state education mandate on the likelihood of completion of the MenACWY vaccine primary and booster dose was diluted by the strong impact of the state booster dose vaccination mandates. The effectiveness of state education mandates for adolescent vaccines remains to be determined.

Among state-level determinants, the pediatricians-to-children ratio was positively associated with the likelihood of completing both vaccine doses. A study by LeBaron et al.^32^ reported a similar finding that this factor was strongly associated with higher vaccination coverage. The proportion of IIS use among adolescents was positively associated with the likelihood of vaccine completion and compliance. The IIS is a computerized database that records all vaccines administered by participating providers within each state. The IIS also assists vaccine providers in assessing appropriate vaccinations for individuals and directs public health resources to improve vaccination coverage.^19,33^

These findings present valuable opportunities for improving vaccine uptake, completion and compliance among adolescents. First, modification of the pediatrician distribution within states may improve MenACWY vaccine primary and booster dose completion. This is in-line with previous observations that more frequent health-care provider contact for the adolescent could potentially reduce missed opportunities for vaccination^30,31^ and improve the likelihood of completing the vaccine series. Second, the implementation of a booster-dose state mandate before the age of 17 is likely to increase vaccination completion. Third, increasing the proportion of state IIS use and state health-care expenditures, especially for services targeted at adolescents, may be promising strategies for enhancing timely receipt of the MenACWY vaccine primary and booster dose.

In the multi-level multivariable regression analyses, 8–9% of the state variation in the odds of MenACWY vaccination could be explained by state-level determinants. While these numbers appear small, they are consistent with ICCs found in other observational studies.^34–36^ In our analyses, the between-state variance in the vaccination outcomes persisted even after adjusting for significant individual-level and state-level determinants. This is demonstrated by the multivariable logistic regression models where the state of residence persisted as an important determinant for completion and compliance. There are several potential explanations for these observations. First, state-level indicators and measures, such as availability of health-care resources, do not capture the granularity that is available at the county- or district-level. Second, delivery methods of vaccination-related mandates vary across states, which was not captured in the list of potential determinants. For example, in the case of education mandates, some states require education from health-care providers while others require programs from the state Department of Health. Studies have found that the influence of education mandates on vaccination rates may depend on the method of delivery.^37^ Third, other state-level determinants, such as state laws on non-physician providers’ ability to vaccinate^38^ or the state methodology for enforcement of mandates, including routes for exceptions, that were not accounted for could have modified or offset the effect of other state-level vaccination policies.^37,39^ Finally, other individual-level characteristics such as individual or parental behaviors, attitudes and beliefs about vaccines, news coverage, provider recommendation, and family influences may also impact vaccination completion and compliance.^40–42^

Several limitations should be considered when interpreting findings from this study. Due to the cross-sectional nature of NIS-Teen data, the analysis did not account for potential individual-level variabilities over time. We were only able to assess characteristics as they were available in the data. It is possible that certain characteristics may change over time, but the potential impact of the dynamic nature of these characteristics could not be captured. Some adolescents may have received vaccinations from different health-care providers and only a subset of these health-care providers may have submitted the provider questionnaire. As such, completion and compliance estimates may be underestimated. Likewise, adolescents who may receive their booster dose after age 17 years are not captured in the NIS-Teen data, which may also contribute to underestimation of the completion rates. Despite the limitations of survey design, this study followed the same methodology as the CDC for estimating vaccine uptake, and estimates were cross-referenced with estimates provided by the CDC whenever possible. Annual estimates in this study were similar to those reported by the CDC. Slight differences may occur due to the exclusion of individuals who received any meningococcal-containing vaccine prior to age 11 years, which was implemented since our study focuses on the ages recommended by ACIP (i.e., starting at age 11 years) and other differences in specific underlying definitions. Combining multiple years of NIS-Teen survey data has its own inherent limitations^25^ but was necessary in this study to enable adequate analyses down to state-level.

In conclusion, although MenACWY vaccine primary and booster dose completion at the appropriate ages and compliance with the ACIP recommendation have improved over time from 2011 to 2016, with significant inter-state variability. This study also identified several individual- and state-level determinants that were significantly associated with completion and compliance with the ACIP recommendation, which may help guide targeted clinical, policy, and educational interventions aimed at improving health-care access/utilization among adolescents. However, after adjusting for both individual-level and state-level characteristics, there remained a persistent effect of the state of residence on the likelihood of MenACWY vaccine completion and compliance. Additional research to elucidate other determinants such as physician or parental behaviors, attitudes and beliefs about vaccines not captured in this study could provide additional insights on optimizing resource allocation and inform efforts for improving MenACWY vaccine completion and compliance rates in US adolescents.

## Abbreviations

ACIP: Advisory Committee on Immunization Practices
AOR: Adjusted odds ratio
CDC: Centers for Disease Control and Prevention
CI: Confidence interval
HPV: Human papillomavirus
ICC: Intraclass correlation coefficient
IIS: Immunization Information System
MenACWY: Quadrivalent meningococcal conjugate vaccine (serogroups A, C, W, and Y
MOR: Median odds ratio
NIS-Teen: National Immunization Survey-Teen
OR: Odds ratio
Tdap: Tetanus, diphtheria, and pertussis
US: United States
