# Supplementary material for: Determinants of Meningococcal ACWY vaccination in adolescents in the US: completion and compliance with the CDC recommendations
Source: Hum Vaccin Immunother. 2019 Aug 16;16(1):176–88. doi: 10.1080/21645515.2019.1632679 (PMC7012109; doi:10.1080/21645515.2019.1632679)
Supplement: Supplemental Material [file khvi-16-01-1632679-s001.zip › Bridging Gaps Supplementary tables.docx]

## Supplementary Table 1: Potential individual-level determinants for MenACWY vaccine completion and compliance

| Variable Description | Variable Name in NIS-Teen | Variable Type |
| --- | --- | --- |
| Adolescent's Demographic Characteristics | | |
| Household survey year | YEAR | Categorical (2011, 2012, 2013, 2014, 2015, 2016) |
| Gender | SEX | Binary (female, male) |
| Race/ethnicity | RACEETHK | Categorical (Hispanic, non-Hispanic White, non-Hispanic Black, non-Hispanic other) |
| Census region | CEN_REG | Categorical (Northeast, Midwest, South, West) |
| State | STATE | Categorical (51 variables, one for each state and DC) |
| Health insurance status | TIS_INS_#  (1, 2, 3, 3A, 4_5, 6), INS_STAT_I | Categorical (private insurance, any Medicaid, other insurance, uninsured) |
| Maternal Characteristics | | |
| Mother's age at time of household survey | AGEGRP_M_I | Categorical (≤34, 35-44, ≥45) |
| Mother's education | EDUC1 | Categorical (<12 years, 12 years, >12 years (no college degree)) |
| Mother's marital status | MARITAL2 | Binary (married, not married) |
| Household Characteristics | | |
| Number of people in household | C1R | Categorical (≤2, 3-4, ≥5) |
| Number of children <18 in household | CHILDNM | Categorical (1, 2-3, ≥4) |
| Poverty status | INCPOV1 | Categorical (below poverty, above poverty ≤$75k, above poverty >$75k) |
| Family income | INCQ298A | Categorical (≤$30k, $30,000-$75k, >$75k) |
| Adolescent's Healthcare History | | |
| Number of visits to healthcare professional in the past year | VISITS | Categorical (none, 1, 2-5, ≥6) |
| Whether teen had a 11-12-year-old well-child exam | CKUP_11_12 | Binary (yes/no) |
| Number of days in past 12 months the teen missed school due to illness/injury | NOSCHOOLR | Categorical (0, 1-5, ≥6) |
| Asthma history | ASTHMA | Binary (yes/no) |
| Chicken pox history | CPOX_HAD | Binary (yes/no) |
| Any high-risk health conditions | RISK_EVER | Categorical (yes/no) |
| Any high-risk health conditions among household members | RISK_HH | Categorical (yes/no) |
| Provider Information | | |
| Number of vaccine providers | N_PRVR | Categorical (one, two, three or more) |
| Facility type of vaccine providers | FACILITY | Categorical (public, private, hospital, other/mixed/unknown) |
| Whether teen's providers report vaccinations to immunization registry | REGISTRY | Categorical (no providers, some providers, all providers, unknown) |
| Whether teen's providers order vaccines from state or local health department | VFC_ORDER | Categorical (no providers, some providers, all providers, unknown) |
| Up-to-date on Other Vaccines |  |  |
| Hepatitis A (2+ hepatitis-A-containing shots) | P_UTDHEPA | Binary (yes/no) |
| Hepatitis B (2+ hepatitis B 1.0 milliliter RECOMBIVAX shots, or 3+ any combination of hepatitis-B-containing shots) | P_UTDHEPB | Binary (yes/no) |
| Measles/mumps/rubella (MMR) (2+ MMR-containing shots) | P_UTDMMR | Binary (yes/no) |
| Varicella (1+ varicella-containing shot at 12+ months of age) | P_UTDVRC | Binary (yes/no) |
| HPV (3+ human papillomavirus shots) | P_UTDHPV3 | Binary (yes/no) |
| Pneumococcal polysaccharide (1+ pneumococcal polysaccharide shot) | P_UTDPPS | Binary (yes/no) |
| MenACWY (for Tdap analysis) (1+ meningococcal-conjugate shot or meningococcal-unknown type shot) | P_UTDMENACWY | Binary (yes/no) |
| Tdap (for MenACWY analysis) (1+ Tdap-only shot since age 10 years) | P_UTDTDAP | Binary (yes/no) |
| Vaccine Mandates | | |
| Residence in a state with one-dose vaccination mandate for MenACWY by age 15 | N/A^a^ | Binary (yes/no) |
| Residence in a state with booster dose vaccination mandate for MenACWY by age 15 | N/A^a^ | Binary (yes/no) |

MenACWY: quadrivalent meningococcal conjugate vaccine against meningococcal disease due to serogroups A, C, W, and Y

Footnotes

^a^This variable was derived from the IAC.

## Supplementary Table 2: Potential state-level determinants for MenACWY vaccine completion and compliance

| Variable Description | Data Source | Variable Type |
| --- | --- | --- |
| Proportion of children ages 0-18 who are on Medicaid | KFF | Binary (above or below US average) |
| Proportion of children ages 0-18 who are uninsured | KFF | Categorical (above US average, below US average, or missing) |
| Proportion of children with a medical home | KFF | Continuous |
| Number of pediatricians per 10,000 population ages 0-18 (i.e., pediatrician density) | KFF | Categorical (based on quartiles) |
| Healthcare expenditures on physician and clinical services per capita | KFF | Continuous |
| Proportion of IIS use among adolescents | CDC | Continuous |
| Residence in a state with education mandate for MenACWY by age 17 | IAC | Binary (yes/no) |

CDC, Center for Disease Control and Prevention; IIS, Immunization Information Systems; IAC, Immunization Action Coalition; KFF, Kaiser Family Foundation; MenACWY: quadrivalent meningococcal conjugate vaccine against meningococcal disease due to serogroups A, C, W, and Y
