# Supplementary material for: Determinants of Meningococcal ACWY vaccination in adolescents in the US: completion and compliance with the CDC recommendations
Source: Hum Vaccin Immunother. 2019 Aug 16;16(1):176–88. doi: 10.1080/21645515.2019.1632679 (PMC7012109; doi:10.1080/21645515.2019.1632679)
Supplement: Supplemental Material [file khvi-16-01-1632679-s001.zip › Supplemetary Fig 1_Bridging gaps.pptx]

## Slide 1
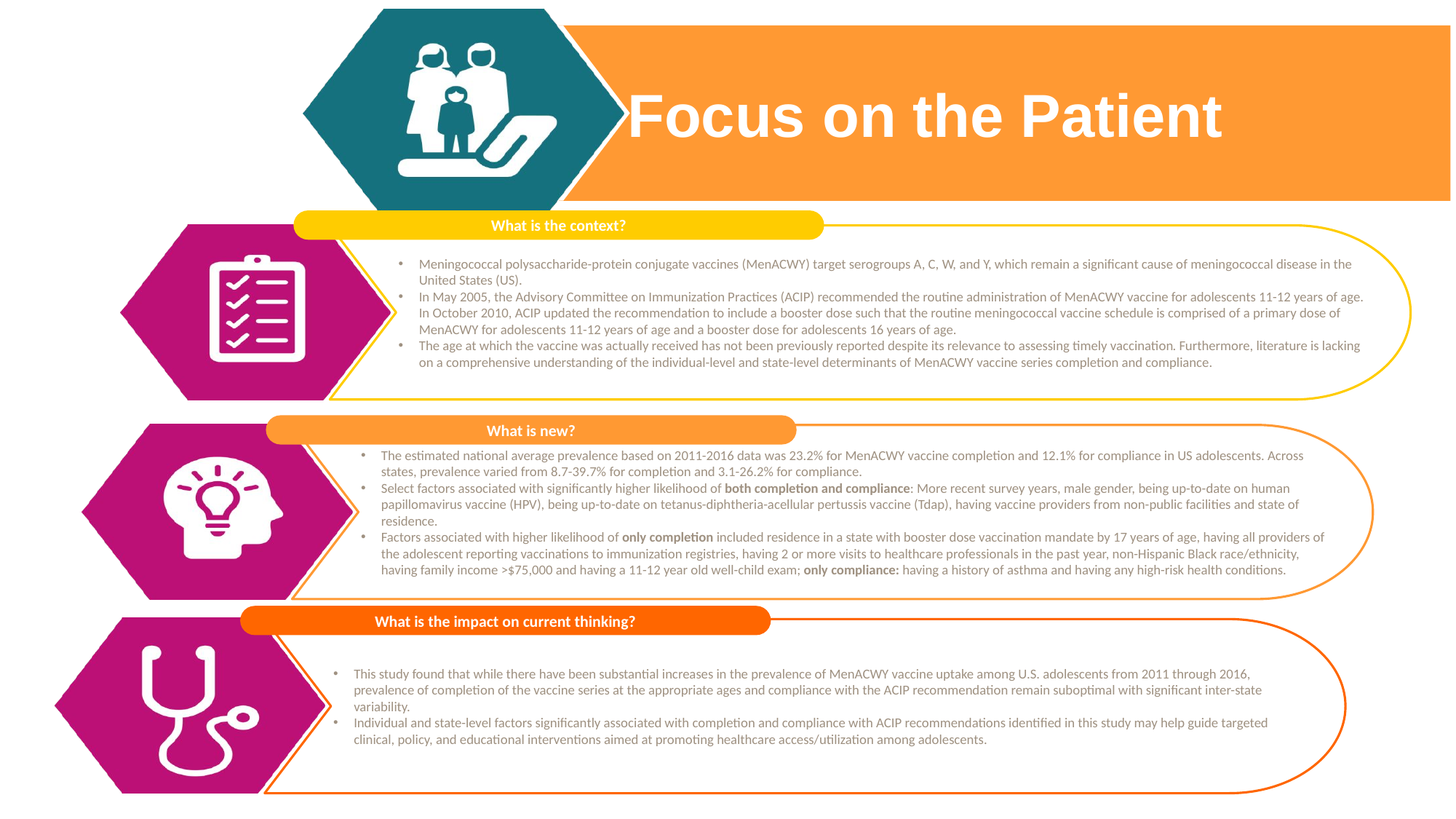

Focus on the Patient
What is the context?
Meningococcal polysaccharide-protein conjugate vaccines (MenACWY) target serogroups A, C, W, and Y, which remain a significant cause of meningococcal disease in the United States (US).
In May 2005, the Advisory Committee on Immunization Practices (ACIP) recommended the routine administration of MenACWY vaccine for adolescents 11-12 years of age. In October 2010, ACIP updated the recommendation to include a booster dose such that the routine meningococcal vaccine schedule is comprised of a primary dose of MenACWY for adolescents 11-12 years of age and a booster dose for adolescents 16 years of age.
The age at which the vaccine was actually received has not been previously reported despite its relevance to assessing timely vaccination. Furthermore, literature is lacking on a comprehensive understanding of the individual-level and state-level determinants of MenACWY vaccine series completion and compliance., as well as compliance with ACIP recommendations
What is new?
The estimated national average prevalence based on 2011-2016 data was 23.2% for MenACWY vaccine completion and 12.1% for compliance in US adolescents. Across states, prevalence varied from 8.7-39.7% for completion and 3.1-26.2% for compliance.
Select factors associated with significantly higher likelihood of both completion and compliance: More recent survey years, male gender, being up-to-date on human papillomavirus vaccine (HPV), being up-to-date on tetanus-diphtheria-acellular pertussis vaccine (Tdap), having vaccine providers from non-public facilities and state of residence.
Factors associated with higher likelihood of only completion included residence in a state with booster dose vaccination mandate by 17 years of age, having all providers of the adolescent reporting vaccinations to immunization registries, having 2 or more visits to healthcare professionals in the past year, non-Hispanic Black race/ethnicity, having family income >$75,000 and having a 11-12 year old well-child exam; only compliance: having a history of asthma and having any high-risk health conditions.
What is the impact on current thinking?
This study found that while there have been substantial increases in the prevalence of MenACWY vaccine uptake among U.S. adolescents from 2011 through 2016, prevalence of completion of the vaccine series at the appropriate ages and compliance with the ACIP recommendation remain suboptimal with significant inter-state variability.
Individual and state-level factors significantly associated with completion and compliance with ACIP recommendations identified in this study may help guide targeted clinical, policy, and educational interventions aimed at promoting healthcare access/utilization among adolescents.
